# Assessment of a Program for SARS-CoV-2 Screening and Environmental Monitoring in an Urban Public School District

**DOI:** 10.1001/jamanetworkopen.2021.26447

**Published:** 2021-09-22

**Authors:** John Crowe, Andy T. Schnaubelt, Scott SchmidtBonne, Kathleen Angell, Julia Bai, Teresa Eske, Molly Nicklin, Catherine Pratt, Bailey White, Brodie Crotts-Hannibal, Nicholas Staffend, Vicki Herrera, Jeramie Cobb, Jennifer Conner, Julie Carstens, Jonell Tempero, Lori Bouda, Matthew Ray, James V. Lawler, W. Scott Campbell, John-Martin Lowe, Joshua Santarpia, Shannon Bartelt-Hunt, Michael Wiley, David Brett-Major, Cheryl Logan, M. Jana Broadhurst

**Affiliations:** 1 Omaha Public School District, Omaha, Nebraska; 2 Department of Neurosurgery, University of Nebraska Medical Center, Omaha; 3 Department of Epidemiology, University of Nebraska Medical Center, Omaha; 4 Department of Environmental, Agricultural, and Occupational Health, University of Nebraska Medical Center, Omaha; 5 Department of Civil Engineering, University of Nebraska Lincoln, Lincoln; 6 Department of Pathology and Microbiology, University of Nebraska Medical Center, Omaha; 7 Department of Medicine, University of Nebraska Medical Center, Omaha; 8 Global Center for Health Security, University of Nebraska Medical Center, Omaha

## Abstract

**Importance:**

Scalable programs for school-based SARS-CoV-2 testing and surveillance are needed to guide in-person learning practices and inform risk assessments in kindergarten through 12th grade settings.

**Objectives:**

To characterize SARS-CoV-2 infections in staff and students in an urban public school setting and evaluate test-based strategies to support ongoing risk assessment and mitigation for kindergarten through 12th grade in-person learning.

**Design, Setting, and Participants:**

This pilot quality improvement program engaged 3 schools in Omaha, Nebraska, for weekly saliva polymerase chain reaction testing of staff and students participating in in-person learning over a 5-week period from November 9 to December 11, 2020. Wastewater, air, and surface samples were collected weekly and tested for SARS-CoV-2 RNA to evaluate surrogacy for case detection and interrogate transmission risk of in-building activities.

**Main Outcomes and Measures:**

SARS-CoV-2 detection in saliva and environmental samples and risk factors for SARS-CoV-2 infection.

**Results:**

A total of 2885 supervised, self-collected saliva samples were tested from 458 asymptomatic staff members (mean [SD] age, 42.9 [12.4] years; 303 women [66.2%]; 25 Black or African American [5.5%], 83 Hispanic [18.1%], 312 White [68.1%], and 35 other or not provided [7.6%]) and 315 students (mean age, 14.2 [0.7] years; 151 female students [48%]; 20 Black or African American [6.3%], 201 Hispanic [63.8%], 75 White [23.8%], and 19 other race or not provided [6.0%]). A total of 46 cases of SARS-CoV-2 (22 students and 24 staff members) were detected, representing an increase in cumulative case detection rates from 1.2% (12 of 1000) to 7.0% (70 of 1000) among students and from 2.1% (21 of 1000) to 5.3% (53 of 1000) among staff compared with conventional reporting mechanisms during the pilot period. SARS-CoV-2 RNA was detected in wastewater samples from all pilot schools as well as in air samples collected from 2 choir rooms. Sequencing of 21 viral genomes in saliva specimens demonstrated minimal clustering associated with 1 school. Geographical analysis of SARS-CoV-2 cases reported district-wide demonstrated higher community risk in zip codes proximal to the pilot schools.

**Conclusions and Relevance:**

In this study of staff and students in 3 urban public schools in Omaha, Nebraska, weekly screening of asymptomatic staff and students by saliva polymerase chain reaction testing was associated with increased SARS-CoV-2 case detection, exceeding infection rates reported at the county level. Experiences differed among schools, and virus sequencing and geographical analyses suggested a dynamic interplay of school-based and community-derived transmission risk. Collectively, these findings provide insight into the performance and community value of test-based SARS-CoV-2 screening and surveillance strategies in the kindergarten through 12th grade educational setting.

## Key Points

**Question:**

Does weekly testing of kindergarten through 12th grade students and staff improve detection of SARS-CoV-2 infection and understanding of the epidemiology of SARS-CoV-2 in urban public school settings?

**Findings:**

In this quality improvement study, weekly school-based saliva polymerase chain reaction testing at 3 urban public schools was associated with increased case detection among staff and students compared with symptom-based strategies, exceeding county-level case rates. SARS-CoV-2 was detected in school wastewater samples each week as well as air and surface samples from choir classrooms.

**Meaning:**

This study suggests that routine SARS-CoV-2 testing may identify infected staff and students who are not identified through conventional case detection and may provide insight into disease burdens of undertested communities.

## Teaser

This quality improvement study characterizes SARS-CoV-2 infections in staff and students in an urban public school setting and evaluates test-based strategies to support ongoing risk assessment and mitigation for kindergarten through 12th grade in-person learning.

## Introduction

During late 2020, most northern hemisphere countries experienced their most severe COVID-19 epidemic waves. Athough many factors may have contributed to the acceleration of SARS-CoV-2 transmission in the fall, the return of minors to congregate school settings was temporally associated with the initiation of increased community transmission.^1^ Overall, direct data demonstrating transmission of SARS-CoV-2 among school-aged children within communities remain limited.^2^ Many experts and public health authorities have urged communities to resume full-time in-person learning, citing the absence of school-associated case clusters and the lower rate of laboratory-confirmed SARS-CoV-2 diagnoses among school-age children vs adults.^3,4,5,6,7^ However, despite the lower rates of confirmed infections among youths, results of serologic surveys suggest that current symptom-based testing and tracing likely miss a large proportion of COVID-19 cases, especially in children and adolescents.^8^ Data through November 2020 from the US Centers for Disease Control and Prevention (CDC) COVID-19 serologic survey revealed that persons aged 17 years or younger had the highest proportion of anti–SARS-CoV-2 antibodies of any age group in 23 of 28 states (82.1%) providing data.^9^ Outbreaks of COVID-19 in schools have been well documented,^10^ including a large outbreak in a school in Israel with infection rates of 13% among students and 17% among staff.^11^

To promote safe school environments, the World Health Organization, CDC, and other health policy centers have recommended combining interventions, such as physical distancing, the wearing of face masks, enhanced environmental cleaning, and the use of physical barriers to reduce the risk of SARS-CoV-2 transmission.^12,13^ Many US school systems have chosen to use virtual or hybrid education models rather than full-density in-person schooling. In developing risk-mitigation plans, public health professionals and school administrators have been forced to weigh incomplete data characterizing COVID-19 school risks with harms of suspending in-person educational services. To make better decisions, school officials require an improved understanding of COVID-19 in the schools and households and greater characterization of in-school transmission risks. In addition, regular testing to rapidly identify students or staff who are at risk for transmitting SARS-CoV-2 may significantly reduce the risk of COVID-19 outbreaks centered in schools, enable more effective learning environments, and ultimately slow community transmission. As SARS-CoV-2 variants expand in the US and the length of protective immunity remains uncertain, these needs persist into the 2021-2022 school year.

The University of Nebraska Medical Center (UNMC) and Omaha Public Schools (OPS) partnered to launch the Proactive Testing for Community Transmission of SARS-CoV-2 (OPS PROTECTS) pilot project in November 2020 to demonstrate the feasibility of a school-based COVID-19 testing program. OPS PROTECTS investigated the integration of individual case detection through saliva testing with school-level wastewater monitoring and in-building air and surface sampling for SARS-CoV-2 RNA.

## Methods

### Pilot Program Setting

The OPS district is composed of 82 primary and secondary schools, roughly 20 programs, and more than 53 000 students and 9200 school-based staff members. Two middle schools and 1 high school were selected to participate in the pilot program, with the goal of maximizing the benefit associated with early program implementation. During the fall 2020 semester, all schools in the district were operating under a hybrid instructional model with alternating cohorts for remote and optional in-person learning, with 50% to 60% of students opting for in-person learning. Districtwide policies to mitigate SARS-CoV-2 transmission risk included mandated in-building face coverings, 1.8-m (6-ft) social distancing in all classrooms and at lunch tables, and heightened handwashing and surface sanitation protocols. The 5-week pilot program took place from November 9 to December 11, 2020. The PROTECTS program received a nonhuman participants research determination by the UNMC institutional review board as an operational public health and quality improvement program. Electronic registration and consent for saliva testing and linkage to clinical services was provided by staff or students’ legal guardians with the Nebraska University Laboratory Information Reporting Tool (NULirt) software platform. Staff participation was compulsory, while student participation was optional. This report follows the revised Standards for Quality Improvement Reporting Excellence (SQUIRE 2.0) reporting guidelines.^14^ SARS-CoV-2 molecular testing and whole-genome sequencing are described in eTable 1 in the Supplement.

### Saliva SARS-CoV-2 Polymerase Chain Reaction Testing for Detection of Asymptomatic Cases

Weekly supervised self-collections of saliva samples were performed at mobile collection stations in each school. Trained volunteers supervised collections according to current CDC guidance.^15^ Saliva samples were collected using half-length plastic straws (S.P. Richards Co) and 1.5-mL collection tubes (Eppendorf Co).

Saliva SARS-CoV-2 testing was performed by the Clinical Laboratory Improvement Amendments (CLIA)–certified Emerging Pathogens Laboratory at UNMC using a multiplex, qualitative, real-time, reverse transcription (RT)–polymerase chain reaction (PCR) assay adapted from the SalivaDirect protocol.^16,17^ Quality monitoring of laboratory testing processes included the sample rejection rate, invalid results rate, and turnaround time for results reporting, in addition to routine quality assurance measures required by the CLIA for high-complexity diagnostic testing. Via NUlirt, test results were securely linked to the electronic medical record and public health databases, and individual results were securely emailed to participants. Individuals with a positive test result were isolated from in-person activities for 10 days and contacted by a health care professional to offer access to clinical services. No further clinical information (ie, development of symptoms or results of tests performed outside the pilot program) was collected.

### School Facility Wastewater Testing for SARS-CoV-2 RNA

Wastewater grab samples (collected at a single point in time) were collected twice weekly (between 11:00 and 13:00) from manholes adjacent to the school buildings. Raw wastewater was collected in sterile 250-mL polypropylene containers. Samples were held at 4 °C for up to 2 hours during transport to UNMC or University of Nebraska Lincoln laboratories and were stored at −20 °C until RT-PCR analysis for SARS-CoV-2 using the IDT 2019-nCoV RUO kit (IDT; 10006713).

### School Building Air and Surface Testing for SARS-CoV-2 RNA

Weekly air and surface sampling was performed in each pilot school at 5 sites per school that were assessed to be at higher risk for virus aerosol exposure, including band and choir rooms, cafeterias, language classrooms, high-traffic hallways, restrooms, and school entry areas. Air samples were collected using AirAnswers (Inspirotec) air samplers,^18^ which collect particles by electrostatic precipitation. Electrostatic precipitation has been used in the collection of a variety of airborne particulates,^19,20^ including viruses.^21^ Surface samples were collected on doors leading into each of the air sampling spaces. Extracted samples were analyzed by RT-PCR for the *SARS-CoV-2 E* gene as previously described.^22^ Further methodological information is in the eMethods in the Supplement.

### Demographic and Statistical Analyses

Metadata associated with SARS-CoV-2 test results included collection location and date, school, status as student or staff, zip code of residence, grade and participation in band or choir for students, and main teaching activity or occupation for staff. The race and ethnicity of participants were self-reported by the individuals based on school enrollment data systems or participants’ registration in NUlirt. Nonpilot program cases among students were identified through school-level designated absentee reports. Staff nonpilot program cases were identified through self-report to the school district. Background community COVID-19 rates were obtained via the Douglas County Health Department, Nebraska online dashboard.^23^ Data handling and analyses were performed in Microsoft Excel 2019, version 2106 (Microsoft Corp) and SAS, version 9.4 (SAS Institute Inc). In addition to descriptive analyses, each metadata element was assessed for association with participants’ first positive test result. Elements were advanced to multivariate logistic regression analysis when an α threshold of .10 was found in bivariate analysis or for presumed confounders. Elements meeting an α threshold of .05 were considered to be statistically significantly associated with the odds of having a positive test result for SARS-CoV-2.

## Results

### Saliva Testing Program Metrics and Participant Demographic Characteristics

Registration and consent numbers for staff and students over the 5-week program period are shown in eFigure 1 in the Supplement. A total of 2885 saliva samples were tested from 773 participants (2163 tests from 458 of 475 school-based staff members [96.4%] [typical staff participant tested 5 times over 5 weeks] and 722 tests from 315 of 2712 students [11.6%] enrolled for in-person learning [typical student participant tested twice over 5 weeks]). Staff participants had a mean (SD) age of 42.9 (12.4) years and were 66.2% female (n = 303), 5.5% Black or African American (n = 25), 18.1% Hispanic (n = 83), 68.1% White (n = 312), and 8.3% other race or not provided (n = 38) (Table). Student participants had a mean (SD) age of 14.2 (0.7) years and were 48% female (n = 151), 6.3% Black or African American (n = 20), 63.8% Hispanic (n = 201), 23.8% White (n = 75), and 6.0% other race (n = 19). More than 77% of student households (244 of 315) were eligible for financial assistance. Almost all saliva samples (2881 of 2899 [99.4%]) met laboratory acceptability criteria and yielded valid test results, with a mean (SD) turnaround time of 4.2 (2.3) hours from receipt in the laboratory to reporting results.

**Table.** Demographic Characteristics of Students and Staff Members in Pilot Schools

Characteristic	Combined students and staff members, No. (%)			
	School A (grades 9-12)	School B (grades 6-8)	School C (grades 5-8)	All program schools
Students eligible for free or reduced-price lunch				
Participants	98/139 (70.5)	61/71 (85.9)	85/105 (81.0)	244/315 (77.5)
Total	1546/1792 (86.3)	1081/1184 (91.3)	1075/1177 (91.3)	3702/4153 (89.1)
Race and ethnicity, students				
Black or African American				
Participants	10/139 (7.2)	5/71 (7.0)	5/105 (4.8)	20/315 (6.3)
Total	225/1792 (12.6)	87/1184 (7.3)	31/1177 (2.6)	343/4153 (8.3)
Hispanic				
Participants	77/139 (55.4)	43/71 (60.6)	81/105 (77.1)	201/315 (63.8)
Total	1220/1792 (68.1)	898/1184 (75.8)	1036/1177 (88.0)	3154/4153 (75.9)
White				
Participants	42/139 (30.2)	16/71 (22.5)	17/105 (16.2)	75/315 (23.8)
Total	242/1792 (13.5)	158/1184 (13.3)	87/1177 (7.4)	487/4153 (11.7)
Other race^a^				
Participants	10 (7.8)	7 (9.9)	2 (1.9)	19 (6.0)
Total	105 (5.7)	41 (3.5)	23 (2.0)	169 (4.1)
Race and ethnicity, staff members^b^				
Black or African American				
Participants	12/179 (6.7)	9/133 (6.8)	4/146 (2.7)	25/458 (5.5)
Hispanic				
Participants	31/179 (17.3)	16/133 (12.0)	36/146 (24.7)	83/458 (18.1)
White				
Participants	118/179 (65.9)	101/133 (75.9)	93/146 (63.7)	312/458 (68.1)
Other race^a^				
Participants	11/179 (6.1)	2/133 (1.5)	8/146 (5.5)	21/458 (4.6)
Not provided				
Participants	7/179 (3.9)	5/133 (3.8)	5/146 (3.4)	17/458 (3.7)

^a^ Includes American Indian or Alaska Native, Asian, Native Hawaiian or Pacific Islander, and 2 or more races.

^b^ Owing to mandatory staff participation in the testing program, participants represent more than 90% of total staff across the pilot schools.

### SARS-CoV-2 Asymptomatic Case Detection by Weekly Saliva Testing

**Figure 1.**
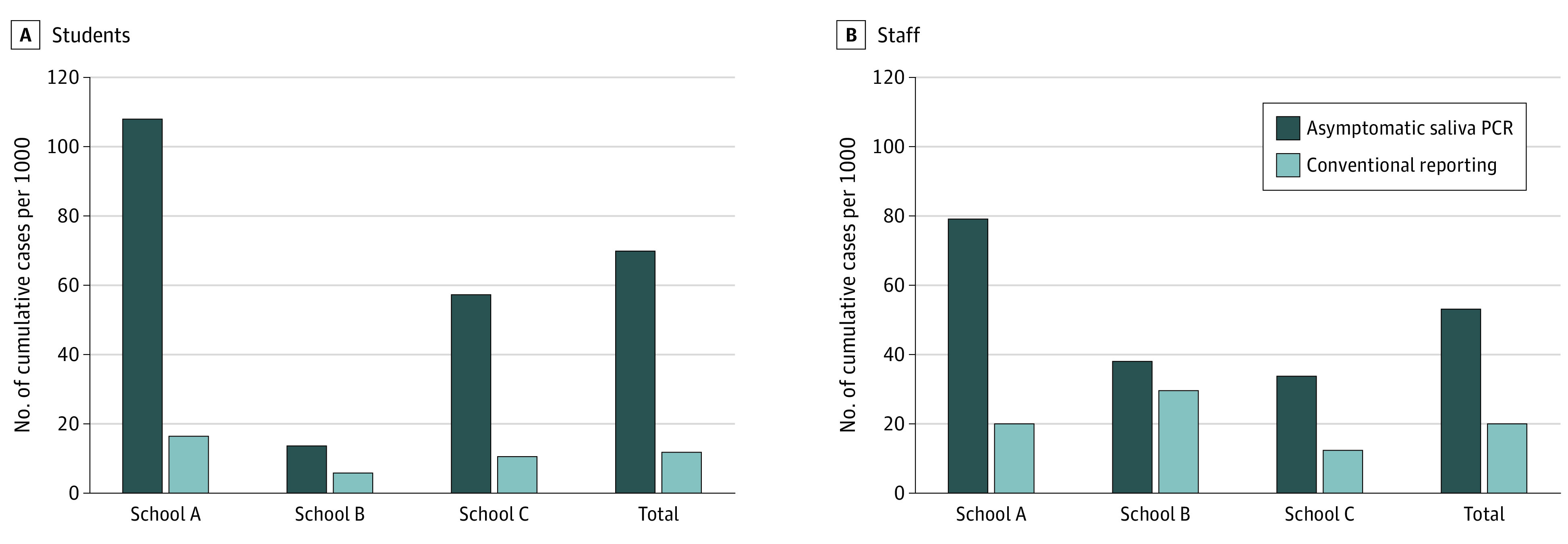
Cumulative SARS-CoV-2 Case Rates Detected by Weekly Saliva Polymerase Chain Reaction (PCR) Testing Compared With Conventional Reporting During the Pilot Program Period For asymptomatic saliva PCR testing, rates are among pilot program participants; for conventional reporting, rates are among individuals participating in in-person school activities.

**Figure 2.**
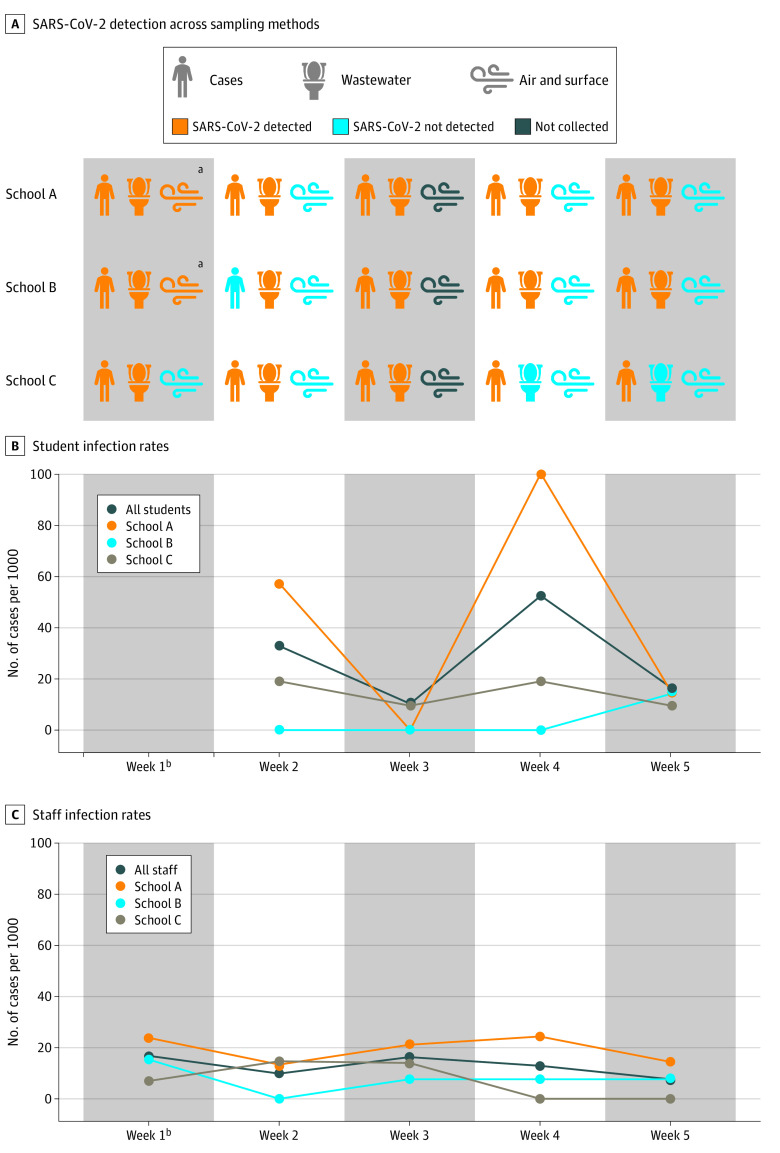
Weekly SARS-CoV-2 Detection by Saliva Testing and Environmental Surveillance Methods Cases represent those detected by weekly saliva PCR testing. ^a^Air and surface samples positive for SARS-CoV-2 RNA were collected in choir rooms. ^b^Staff saliva testing began during week 1; student saliva testing began during week 2.

A total of 46 cases of COVID-19 (24 staff members and 22 students) were detected through weekly saliva PCR testing of asymptomatic individuals. The pilot program was associated with more than double the number of cases identified among staff members and nearly double the number of cases identified among students (eFigure 2 in the Supplement). Cumulative case rates detected by saliva testing among pilot program participants substantially exceeded case rates detected by conventional reporting mechanisms among students and staff members registered for in-person school activities during the same time period at the pilot schools (students, 70 of 1000 [7.0%] vs 12 of 1000 [1.2%]; staff members, 53 of 1000 [5.3%] vs 21 of 1000 [2.1%]; Figure 1). Weekly case detection rates by saliva PCR across all pilot schools ranged from 10 to 52 cases per 1000 students (1.0%-5.2%) and 8 to 17 cases per 1000 staff members (0.8%-1.7%) (Figure 2). Most staff cases were detected within 1 week from their last negative test result, while half of the student cases were detected at 2 to 3 weeks from their last negative test result, indicating less-consistent student participation (eFigure 3 in the Supplement). Fifteen of 17 individuals with positive PCR test results (88.2%) who participated in retesting had negative results on their first test after a 10-day isolation period.

### SARS-CoV-2 RNA Detection in School Facility Wastewater and In-Building Environmental Samples

SARS-CoV-2 RNA was detected by RT-PCR in wastewater grab samples from school A and school B facilities during all 5 weeks of the pilot program and at school C for the first 3 weeks of the pilot program (Figure 2). Weekly wastewater testing detected SARS-CoV-2 in 12 of 14 individual school-weeks (85.7%) for which SARS-CoV-2 was detected in participants by saliva testing.

In-building air and surface samples were collected from 5 sampling sites in each pilot school during 4 weeks of the pilot program (60 total paired samples). A subset of air samples (2 of 60 [3.3%]) and surface samples (1 of 60 [1.7%]) had positive results for SARS-CoV-2 RNA by RT-PCR; all were collected from choir rooms during the first week of the pilot program (Figure 2; eTable 2 in the Supplement). Choir room surfaces were subsequently sanitized, and saliva testing for choir staff was conducted.

### Virus Whole-Genome Sequencing of SARS-CoV-2–Positive Saliva Samples

**Figure 3.**
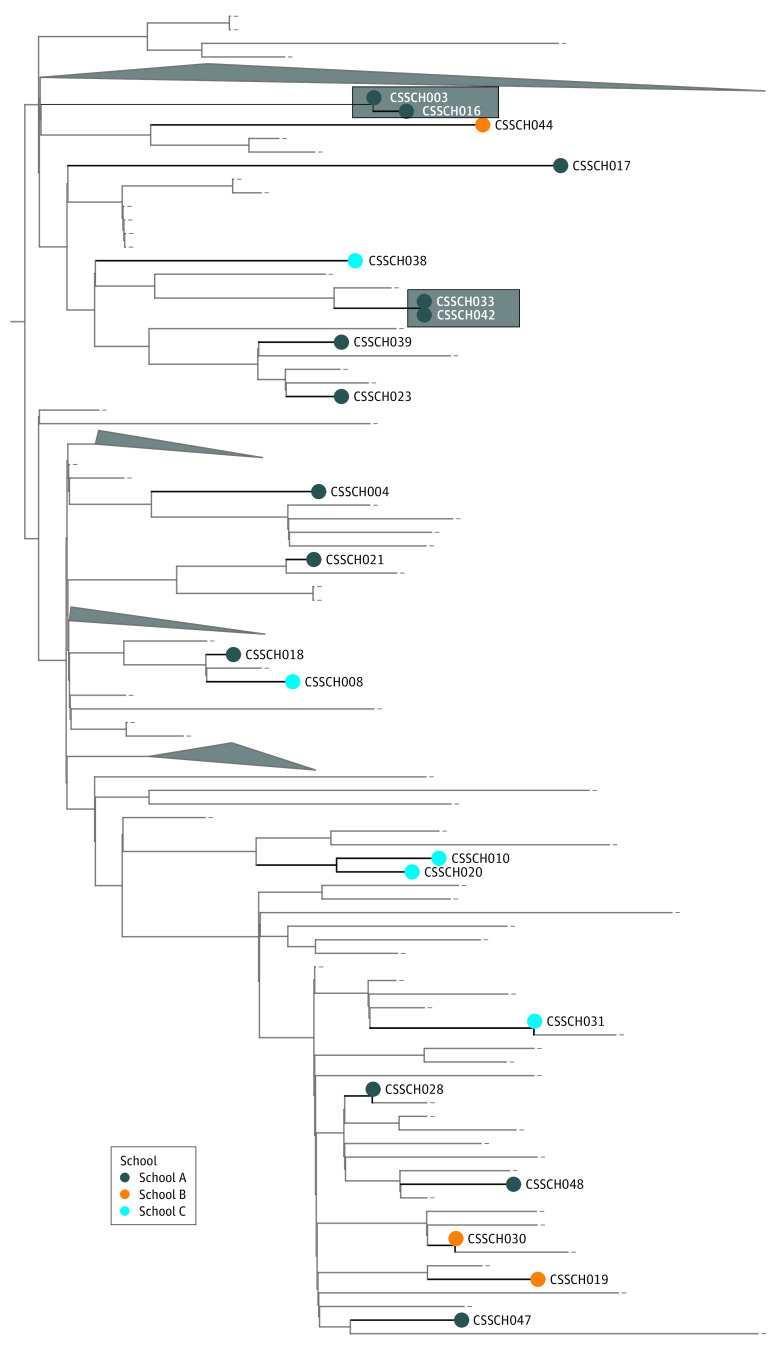
Phylogenetic Analysis of SARS-CoV-2 Sequences From Saliva Specimens Collected at Pilot Schools Clustered samples are shaded in gray.

SARS-CoV-2 genomes from positive saliva samples were sequenced to investigate transmission chains within cases identified by our testing program. We obtained high-coverage viral genomes from 21 of 46 cases and generated phylogenetic trees using the available Nebraska SARS-CoV-2 genomes from the Global Initiative on Sharing All Influenza Data (GISAID) (177 genomes). Most SARS-CoV-2 genomes (17 of 21) did not cluster together but were widely spread across the phylogenetic tree, indicating that these cases were the result of separate transmission chains (Figure 3). We observed 2 clustering events with samples from school A (highlighted in Figure 3), between 2 staff members (CSSCH003 and CSSCH016, identical genomes) and 2 students (CSSCH033 and CSSCH042, 1 nucleotide difference). Clear epidemiologic links between the clustered genomes beyond school attendance were not identified; however, the timing of case identification was compatible with linked transmission events.

### School-Based Risk Factors for SARS-CoV-2 Infection

Participation in school activities, school attended, school grade, and staff position were evaluated as possible risk factors for SARS-CoV-2 infection as detected by saliva PCR testing during the pilot program (eTable 3 in the Supplement). School attended was significantly associated with positive test results in logistic regression models. School A (high school) students and staff members were more likely than those at schools B and C (middle schools) to test positive (students: odds ratio, 3.3 [95% CI, 1.3-8.1]; *P* = .009; staff members: odds ratio, 2.2 [95% CI, 1.1-4.5]; *P* = .03). Case rates at schools B and C were similar.

Students in choir were 2.8 times (95% CI, 0.99-8.1) as likely to test positive for SARS-CoV-2 than other students when adjusting for school attended (*P* = .05); in unadjusted odds, students in choir were 1.9 times (95% CI, 0.74-4.9; *P* = .19) as likely to test positive for SARS-CoV-2 than other students. The impact of choir did not reach statistical significance. Case amplification over serial weeks among students participating in choir was not observed (eFigure 4 in the Supplement).

Broadly, the category of staff position was not significantly associated with SARS-CoV-2 testing results. However, business teachers in the pilot program (n = 7) were 28.5 (95% CI, 6.0-136.0) times as likely to test positive for SARS-CoV-2 than other staff members (*P* < .001). They were at more than 1 school.

### Geographical Distribution of District-Wide COVID-19 Cases Reported Among Staff and Students

**Figure 4.**
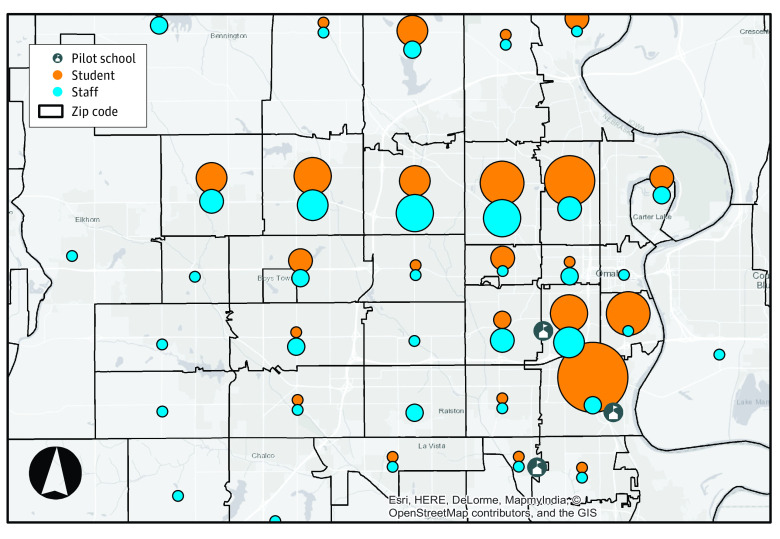
Geographical Distribution of Home Residence Zip Codes Linked to COVID-19 Cases Reported for Staff and Students Across the School District This graphic depicts the district-wide COVID-19 case burden by zip code of residence; the position of circles within each zip code is arbitrary. Circle size denotes relative number of cases reported by zip code. This graphic was generated employing OPS PROTECTS (Proactive Testing for Community Transmission of SARS-CoV-2) data atop Esri layers World Hillshade and World Topographic Map, under the Esri master agreement.^24,25^

Weekly case rates detected by saliva testing in the pilot schools exceeded county-level case rates identified through conventional testing means over the same time period by a log. We sought to better understand the role of localized community case burden in these findings. In preliminary bivariate analyses, zip code of residence did not achieve statistical significance as a factor associated with saliva SARS-CoV-2 test result. To contextualize the pilot schools within the district, Figure 4^24,25^ demonstrates the distribution of cases by zip code of residence for students and staff members across the entire OPS district, through either self-report or district-managed occupational screening separate from the pilot program. Student case counts were highest in a South Omaha zip code, with 66 cases during the pilot period, demonstrating a higher case burden in the community sector where the pilot schools are located.

## Discussion

OPS PROTECTS represents a feasible, scalable, and novel approach to test-based SARS-CoV-2 screening and environmental monitoring in a kindergarten through 12th grade educational setting. Compared with conventional reporting in our setting (passive case finding), our results suggest that as many as 9 in 10 student COVID-19 cases and 7 in 10 staff COVID-19 cases may be missed by conventional reporting mechanisms. SARS-CoV-2 testing was freely available in the Omaha metropolitan area at the time of the pilot, with a mean test positivity rate during the pilot program that suggests low uptake by the public (15% in Nebraska and 32% in Douglas County).^23,26^ The differences between the pilot program case rates and observed community case rates indicate that programs such as OPS PROTECTS may assist in mitigating school-based transmission risk through informing case isolation, contact tracing, and management of school activities and may serve as a trigger to escalate community-based surveillance, particularly among school-aged children.

To our knowledge, this study represents the first description of building-level environmental testing for SARS-CoV-2 in kindergarten through 12th grade schools. During the OPS PROTECTS pilot program, wastewater was tested for SARS-CoV-2 alongside weekly saliva samples to evaluate the utility of wastewater testing for school building–level surveillance. Wastewater monitoring for SARS-CoV-2 RNA by twice-weekly grab sampling was generally consistent with the detection of SARS-CoV-2 infections by saliva testing; however, 2 wastewater collections (school C, weeks 4 and 5) yielded negative results while the schools still had persons with positive saliva test results. The use of wastewater autosampling instruments for the collection of time-distributed composite wastewater samples may increase sensitivity for case detection and will be further explored. Considering all of the costs for collection and testing, as well as laboratory processing and analysis, the cost of twice-weekly PCR testing of composite wastewater samples was approximately $750 per week per school, which equates to $0.75 per person for a midsize school of 1000 students and staff members compared with typical costs of $10 to $50 per person for the screening of pooled or individual clinical samples. Thus, wastewater and other environmental monitoring may ultimately provide cost-effective, building-level surveillance to identify SARS-CoV-2 transmission hotspots and prioritize more resource-intensive individual screening, a strategy that has been successfully pursued in university housing^27,28^ and nursing home^29^ settings.

Air and surface sampling within school buildings for SARS-CoV-2 RNA provides insight into virus dispersion associated with school activities and environments. This information may assist in the evaluation of activity-specific transmission risk and inform risk mitigation measures, including activity modifications and enhanced hygiene protocols. In our pilot program, air and surface samples with positive results for SARS-CoV-2 were detected in the choir rooms of 2 schools during the first week of the program. These findings suggest that measures to reduce the level of virus dispersion in the school environment did not fully mitigate the risk associated with singing during choir class. Choir-associated COVID-19 outbreaks have been described,^30,31^ and we observed a trend toward increased risk of SARS-CoV-2 infection among students participating in the choir. Our initial findings regarding business teachers suggest a higher risk for infection among teachers associated with computer laboratories.

### Limitations

This study has some limitations. Although our findings confirm that the incidence of SARS-CoV-2 infection in schools greatly exceeded what was observed through conventional case findings, our data do not permit firm conclusions about comparative incidence or transmission events within schools. Genomic sequencing identified potential transmission links among students and staff members in 2 clusters at school A, but the dectection of the virus from saliva samples in our pilot demonstrated mostly a mix of multiple disparate transmission chains compatible with a broader community transmission. Our highest weekly incidence for students (week 4) was more than 7 times the reported weekly community incidence for Douglas County (week of November 30), and the incidence in school A was 14-fold higher. However, our schools are located in communities that experienced higher incidence and where limited testing access may accentuate underascertainment of cases. Our schools were also operating at one-fourth normal classroom densities, so our results may underestimate the risk of in-school transmission for schools operating at more normal density.

Student participation in the pilot program was likely associated with multiple factors. One-third of the students who received parental consent for testing were excluded from participation owing to declined consent for treatment required by the ordering clinician (eFigure 1 in the Supplement). Other factors that may have been negatively associated with student participation include technical barriers to digital registration and consent and the requirement for home isolation after positive test results. Higher student participation would allow for a better understanding of case rates within schools, demographic risk groups among students and staff members, and correlation with wastewater and in-building environmental testing strategies. The time intervals for test conversion indicate that promoting consistent student participation, in addition to increasing student consent rates, will improve the timely detection and isolation of new cases.

## Conclusions

In this study of staff and students in 3 urban public schools in Omaha, Nebraska, weekly screening of asymptomatic staff and students by saliva PCR testing was associated with increased SARS-CoV-2 case detection, exceeding infection rates reported at the county level. Experiences differed among schools, and virus sequencing and geographical analyses suggested a dynamic interplay of school-based and community-derived transmission risk. Collectively, these findings provide insight into the performance and community value of test-based SARS-CoV-2 screening and surveillance strategies in the kindergarten through 12th grade educational setting.

## Notes

Supplement.
eMethods.
**eTable 1.** Acknowledgment of Contributors to GISAID SARS-CoV-2 Genome Sequences Used in This Study **eTable 2.** SARS-CoV-2 RNA Detection in School Building Air and Surface Samples **eTable 3.** Demographic Risk Factors for SARS-CoV-2 Infection in Pilot Participants **eFigure 1.** Cumulative Registrations and Consents Over the Pilot Program Period **eFigure 2.** Incremental Surveillance Value of Asymptomatic Saliva Testing in the Pilot Program **eFigure 3.** Time Interval for SARS-CoV-2 Saliva PCR Test Conversions **eFigure 4.** Weekly SARS-CoV-2 Case Detection by Saliva PCR Among Choir Students in Each Pilot School

## References

[1] Chernozhukov V, Kasahara H, Schrimpf P. The association of opening K-12 schools and colleges with the spread of COVID-19 in the United States: county-level panel data analysis. *medRxiv*. Preprint posted online February 23, 2021. doi:10.1101/2021.02.20.21252131 PMC8545468 34642247

[2] Brauner JM, Mindermann S, Sharma M, . Inferring the effectiveness of government interventions against COVID-19. Science. 2021;371(6531):eabd9338. doi:10.1126/science.abd9338 33323424 PMC7877495

[3] Ismail SA, Saliba V, Lopez Bernal J, Ramsay ME, Ladhani SN. SARS-CoV-2 infection and transmission in educational settings: a prospective, cross-sectional analysis of infection clusters and outbreaks in England. Lancet Infect Dis. 2021;21(3):344-353. doi:10.1016/S1473-3099(20)30882-3 33306981 PMC7833602

[4] Buonsenso D, De Rose C, Moroni R, Valentini P. SARS-CoV-2 infections in Italian schools: preliminary findings after 1 month of school opening during the second wave of the pandemic. Front Pediatr. 2021;8:615894. doi:10.3389/fped.2020.615894 33520898 PMC7841339

[5] Leidman E, Duca LM, Omura JD, Proia K, Stephens JW, Sauber-Schatz EK. COVID-19 trends among persons aged 0-24 years—United States, March 1-December 12, 2020. MMWR Morb Mortal Wkly Rep. 2021;70(3):88-94. doi:10.15585/mmwr.mm7003e1 33476314 PMC7821770

[6] Zimmerman KO, Akinboyo IC, Brookhart MA, ; ABC SCIENCE COLLABORATIVE. Incidence and secondary transmission of SARS-CoV-2 infections in schools. Pediatrics. 2021;147(4):e2020048090. doi:10.1542/peds.2020-048090 33419869 PMC8015158

[7] Falk A, Benda A, Falk P, Steffen S, Wallace Z, Høeg TB. COVID-19 cases and transmission in 17 K-12 schools—Wood County, Wisconsin, August 31-November 29, 2020. MMWR Morb Mortal Wkly Rep. 2021;70(4):136-140. doi:10.15585/mmwr.mm7004e3 33507890 PMC7842817

[8] Ng OT, Marimuthu K, Koh V, . SARS-CoV-2 seroprevalence and transmission risk factors among high-risk close contacts: a retrospective cohort study. Lancet Infect Dis. 2021;21(3):333-343. doi:10.1016/S1473-3099(20)30833-1 33152271 PMC7831879

[9] Centers for Disease Control and Prevention. COVID data tracker. Accessed February 16, 2021. https://covid.cdc.gov/covid-data-tracker

[10] Gold JAW, Gettings JR, Kimball A, ; Georgia K–12 School COVID-19 Investigation Team. Clusters of SARS-CoV-2 infection among elementary school educators and students in one school district—Georgia, December 2020-January 2021. MMWR Morb Mortal Wkly Rep. 2021;70(8):289-292. doi:10.15585/mmwr.mm7008e4 33630823 PMC8344983

[11] Stein-Zamir C, Abramson N, Shoob H, . A large COVID-19 outbreak in a high school 10 days after schools’ reopening, Israel, May 2020. Euro Surveill. 2020;25(29). doi:10.2807/1560-7917.ES.2020.25.29.2001352 32720636 PMC7384285

[12] Centers for Disease Control and Prevention. Community, work, & school: update. Accessed February 16, 2021. https://www.cdc.gov/coronavirus/2019-ncov/community/schools-childcare/index.html

[13] Global Center for Health Security, University of Nebraska Medical Center. Industry playbooks. Accessed February 22, 2021. https://www.unmc.edu/healthsecurity/covid-19/playbooks/index.html

[14] SQUIRE. Revised standards for quality improvement reporting excellence: SQUIRE 2.0. Accessed May 5, 2021. http://squire-statement.org/index.cfm?fuseaction=Page.ViewPage&PageID=471

[15] Centers for Disease Control and Prevention. Interim guidelines for collecting and handling of clinical specimens for COVID-19 testing. Accessed February 16, 2021. https://www.cdc.gov/coronavirus/2019-ncov/lab/guidelines-clinical-specimens.html

[16] Vogels C, Brackney DE, Chaney CK, Ott IM, Grubaugh N, Wyllie A. SalivaDirect: RNA extraction-free SARS-CoV-2 diagnostics V.5. Published online September 1, 2020. Accessed September 1, 2020. https://www.protocols.io/view/salivadirect-rna-extraction-free-sars-cov-2-diagno-bkjgkujw?version_warning=no

[17] Vogels CBF, Watkins AE, Harden CA, ; Yale IMPACT Research Team. SalivaDirect: a simplified and flexible platform to enhance SARS-CoV-2 testing capacity. Med (N Y). 2021;2(3):263-280.e6. doi:10.1016/j.medj.2020.12.010 33521748 PMC7836249

[18] Gordon J, Gandhi P, Shekhawat G, Frazier A, Hampton-Marcell J, Gilbert JA. A simple novel device for air sampling by electrokinetic capture. Microbiome. 2015;3:79. doi:10.1186/s40168-015-0141-2 26715467 PMC4696304

[19] Gordon J, Reboulet R, Gandhi P, Matsui E. Validation of a novel sampling technology for airborne allergens in low-income urban homes. Ann Allergy Asthma Immunol. 2018;120(1):96-97. doi:10.1016/j.anai.2017.10.002 29273138 PMC6333298

[20] Richardson M, Gottel N, Gilbert JA, . Concurrent measurement of microbiome and allergens in the air of bedrooms of allergy disease patients in the Chicago area. Microbiome. 2019;7(1):82. doi:10.1186/s40168-019-0695-5 31159879 PMC6547563

[21] Kettleson EM, Ramaswami B, Hogan CJ Jr, . Airborne virus capture and inactivation by an electrostatic particle collector. Environ Sci Technol. 2009;43(15):5940-5946. doi:10.1021/es803289w 19731701

[22] Santarpia JL, Rivera DN, Herrera VL, . Aerosol and surface contamination of SARS-CoV-2 observed in quarantine and isolation care. Sci Rep. 2020;10(1):12732. doi:10.1038/s41598-020-69286-3 32728118 PMC7391640

[23] Douglas County Health Department. Douglas County NE COVID-19 dashboard. Accessed February 16, 2021. https://experience.arcgis.com/experience/1205c60366ba43719a59225ec62e31b5

[24] ArcGIS. World hillshade—overview. Accessed May 9, 2021. https://www.arcgis.com/home/item.html?id=1b243539f4514b6ba35e7d995890db1d

[25] ArcGIS. World topographic map—overview. Accessed May 9, 2021. https://www.arcgis.com/home/item.html?id=30e5fe3149c34df1ba922e6f5bbf808f

[26] Nebraska COVID-19 dashboards. Accessed May 9, 2021. https://experience.arcgis.com/experience/ece0db09da4d4ca68252c3967aa1e9dd

[27] Betancourt WW, Schmitz BW, Innes GK, . Wastewater-based epidemiology for averting COVID-19 outbreaks on the University of Arizona campus. Preprint. Posted November 16, 2020. medRxiv. doi:10.1101/2020.11.13.20231340

[28] Gibas C, Lambirth K, Mittal N, . Implementing building-level SARS-CoV-2 wastewater surveillance on a university campus. Sci Total Environ. 2021;782:146749. doi:10.1016/j.scitotenv.2021.146749 33838367 PMC8007530

[29] Davó L, Seguí R, Botija P, . Early detection of SARS-CoV-2 infection cases or outbreaks at nursing homes by targeted wastewater tracking. Clin Microbiol Infect. 2021;27(7):1061-1063. doi:10.1016/j.cmi.2021.02.003 33601008 PMC7882920

[30] Charlotte N. High rate of SARS-CoV-2 transmission due to choir practice in france at the beginning of the COVID-19 pandemic. J Voice. Published online December 23, 2020. doi:10.1016/j.jvoice.2020.11.029 33386191 PMC7833901

[31] Hamner L, Dubbel P, Capron I, . High SARS-CoV-2 attack rate following exposure at a choir practice—Skagit County, Washington, March 2020. MMWR Morb Mortal Wkly Rep. 2020;69(19):606-610. doi:10.15585/mmwr.mm6919e6 32407303

